# Iloprost in Acute Post-kidney Transplant Atheroembolism: A Case Report of Two Successful Treatments

**DOI:** 10.3389/fmed.2020.00041

**Published:** 2020-02-28

**Authors:** Valeria Corradetti, Giorgia Comai, Matteo Ravaioli, Vania Cuna, Valeria Aiello, Federica Odaldi, Andrea Angeletti, Irene Capelli, Gaetano La Manna

**Affiliations:** ^1^Department of Experimental Diagnostic and Specialty Medicine (DIMES), Nephrology, Dialysis and Renal Transplant Unit, St. Orsola Hospital, University of Bologna, Bologna, Italy; ^2^Unit of General and Transplant Surgery, Department of Medical and Surgical Sciences, University of Bologna, S. Orsola Malpighi Hospital Bologna, Bologna, Italy

**Keywords:** cholesterol embolism, kidney transplant, prostaglandin agonism, delayed graft function, extended criteria donors

## Abstract

Cholesterol embolization (CE) is a rare and alarming post-transplant complication, responsible for primary non-function (PNF) or delayed graft function (DGF). Its incidence is expected to rise due to increasingly old donors and recipients and the extended criteria for donation. Therapy with statins and steroids has not been shown to be effective, while agonism of prostaglandin I_2_ has been reported to be useful in systemic CE. We report two cases of acute post-transplant CE in which intravenous iloprost (0.05 mg/kg/day) was added to standard statin and steroid therapy. In the first instance, CE was due to embolization from the kidney artery resulting in embolization of the small vessels; after a long DGF and 15 days of iloprost therapy, renal function recovered. The second instance is a case of embolization from the iliac artery of the recipient, where CE manifested as a partial renal infarction. After 5 days of iloprost administration, creatinine levels improved. Iloprost acts on vasodilation and on different inflammatory pathways, improving the anti-inflammatory profile. Post-transplant CE is difficult to diagnose and, if not treated, can lead to loss of function. Iloprost added to standard therapy could be beneficial in accelerating renal function recovery immediately after transplant.

## Introduction

Cholesterol embolization (CE) is a rare but alarming complication in renal allograft. Its reported frequency is roughly 0.4% (1–3) and, when it presents acutely after transplant, is recognized as one of the causes of primary non-function (PNF) and delayed graft function (DGF) (2, 4, 5).

Considering the increase in transplants from extended criteria donors (ECDs), from donation after circulatory death (DCD), and the tendency for recipients to be older, the possibility of embolization arising from either donor or recipient vessels is expected to increase (6–10). Moreover, since embolization leads to focal and patchy damage, diagnosis is difficult, and injury severity may be underestimated (2, 11–13).

In the absence of a standard and effective therapy, strategies usually aim at stabilizing the plaque by using statins associated with steroids if the disease is recurring and systemic. Reports describe the effectiveness of iloprost, a synthetic analog of prostaglandin I_2_, as a rescue therapy in systemic CE (2, 11, 14–16). Moreover, in the coronary angiography setting, where ischemic damage to renal tissue is the leading pathogenic mechanism, a reduction in the incidence of contrast-induced nephropathy has been reported in patients with baseline renal insufficiency undergoing coronary intervention (17).

To the best of our knowledge, there are no recent reports on the use of iloprost in CE after kidney transplantation (2, 16, 18).

Here we report two cases of acute post-transplant CE in which the addition of iloprost to the standard care helped accelerate the recovery of kidney function.

Written informed consent was obtained from the participants for the publication of these case reports.

## Case Report

### Case 1

**Figure 1:**
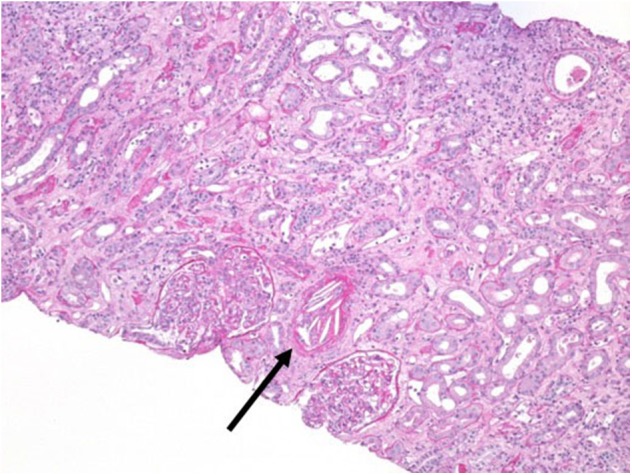
Kidney biopsy at post-operative day 16. Periodic acid–Schiff (PAS) staining, magnification 20×. Arrow indicates a massive cholesterol embolization occluding the arteriolar lumen.

A 44-year-old man received a kidney transplant from a brain-dead donor (DBD). The donor was 59 years old, had died from cerebral hemorrhage, his Kidney Donor Profile Index (KDPI) was 83%, Karpinsky's score was 3, and he had been a smoker with a past history of prostate cancer for which he was in regular follow-up (19). The surgeon described atheromatous plaques in the renal artery that were particularly evident at the confluence with the aorta and were partially removed before implantation. Immunosuppressive therapy consisted of basiliximab, steroids, and tacrolimus. Owing to persistent oligo-anuria, a kidney biopsy was performed on the eighth post-operative day (POD). Histology showed severe acute tubular necrosis (ATN), diffuse cholesterol embolism in the arterioles, inflammatory mixed infiltrate, and interstitial edema. A borderline cellular rejection was diagnosed, and thymoglobulin (ATG) therapy at a dose of 3 mg/kg was administered. Because of the persistence of DGF, the kidney biopsy was repeated on POD 16. The sample showed regression of the interstitial infiltrate, with persistence of ATN and diffuse CE (Figure 1). Therefore, we started rescue therapy with intravenous iloprost at a dose of 0.05 mg/kg/day for 15 days. We observed a slow but progressive recovery of kidney function. No peripheral signs of embolism were observed on physical examination. After 3 months, creatinine was 3 mg/dl; at the 1 year of follow-up, it had improved to 2 mg/dl (Table 1). In this case, the probable source of embolization was the donor renal artery, which presented as a severe atherosclerotic plaque at retrieval.

**Table 1:** Clinical course of both cases.

	**Case 1**				**Case 2**			
	**Post-surgery**	**Pre-iloprost**	**During iloprost**	**After iloprost**	**Post-surgery**	**Pre-iloprost**	**During iloprost**	**After iloprost**
Blood pressure (mmHg)		130/65	130/80	120/80	110/70	140/85	130/70	140/80
Urine volume (ml/day)	0	0	1,200	2,000	1,500	1,000	1,200	1,200
Creatinine (mg/dl)	8	8.2	8	3	1.4	2.4	2	1.6
Urea (mg/dl)	118	169	157	90	54	78	81	73
Eosinophils 10^9^/L	0.14	0	0.15	0.07	0.06	0.16	0.22	0.09
LDH U/L	357	302	349	250	370	1193	540	402

### Case 2

A 71-year-old hypertense woman underwent a DBD double kidney transplant. The iliac vessels of the recipient, a smoker, presented with severe atheromatous plaques such that it was difficult to find a suitable vessel to perform the arterial anastomoses; some plaques were fixed to the walls of the vessel with 6-0 prolene. The 81-year-old donor had died from a cerebral hemorrhage, had a KDPI of 99%, and a Karpinsky's score of 4 in both kidneys. Immunosuppressive therapy consisted of ATG, steroids, and tacrolimus. The graft function was prompt, with creatinine levels of 1.7 mg/dl on POD 4, and routine ultrasounds were normal. On POD 13, we observed an abrupt rise in creatinine (2.4 mg/dl), lactate dehydrogenase (LDH) 1,100 U/l, and a slight decrease in diuresis. A contrast-enhanced ultrasonography showed a lack of vascularization in the upper pole of one of the kidneys compatible with a partial infarction. Intravenous iloprost at a dose of 0.05 mg/kg/day was administered as a rescue therapy for 5 days. After 3 days, we started to see progressive recovery of kidney function; after 3 months, the creatinine level was 1.5 mg/dl (Table 1). No peripheral signs of embolism were observed on physical examination. In this case, the most likely source of embolization was the recipient's iliac artery.

## Discussion

Atheroembolic renal disease in kidney transplantation is recognized as a possible cause of graft loss. It can occur in the early days post-transplant as well as in the late phases of transplant follow-up (2, 4, 5).

When presenting acutely post-transplant, CE usually occurs due to an acute embolization from either the aorta or the renal artery of the donor during organ harvesting or from the vascular axis of the recipient during surgery.

As a result of the increasing number of ECD and of the aging of both donor and recipient population, atherosclerosis of the vascular axis of the graft and of the recipient is becoming a serious challenge in the field of organ transplantation (20–23).

In our first case, we described the embolization of the donor artery in which plaque disruption probably occurred at harvesting or during the preliminary vascular manipulation made before implantation. In the second instance, the likely cause the acute deterioration of function was crystal embolization from the recipient iliac artery. Our final diagnosis was difficult to prove since no peripheral or systemic signs of CE were present, no other causes of acute kidney injury were identified, and an ischemic area was clearly identified by contrast-enhanced ultrasound. We were aware that the patient was severely atherosclerotic from the results of multiple computed tomography-angiographies performed during the time spent on the waiting list. The surgeon, due to our experience in high atherosclerotic patients, defined her to be suitable for transplant; her condition, however, was found to be worse than predicted.

When the plaque disrupts, microemboli spray downstream, and occlude the vascular lumen of small arteries. The ensuing damage is a combination of tissue ischemia, direct cytotoxic effects of crystals, and necrosis due to the local inflammatory reaction. Soon after embolization, the first damage occurs to endothelium mitochondria (24). Then, because of the large dimensions of cholesterol crystals (1 μm−1 mm), macrophages are not able to digest them completely; this “*frustrated phagocytosis*” triggers an intracellular danger signal mediated by damage-associated molecular patterns, interleukin (IL)-1α, IL-1β, and nuclear factor (NF)-κB (24–27). The vicious cycle of necroinflammation eventually leads to necrosis (28, 29). Moreover, it has been demonstrated that cholesterol crystals also activate the complement-dependent inflammasome and cytokines (30, 31). Overall, the ischemic and inflammatory pathways activated by this phenomenon increase the already high cardiovascular and inflammatory risk profile of transplant recipients (32).

Since the damage caused by CE is patchy, it is well-known that histologic diagnosis is difficult and often underestimated; this also occurs in native kidneys (12, 13). Moreover, cholesterol crystals are not always present in the sample, and the only lesions seen are ATN and inflammatory infiltrates (2). In light of this, the mild interstitial mixed infiltrate already present in the biopsy of our first case could be explained as related more to an inflammatory reaction to the severe and diffuse embolism rather than to cellular rejection, especially considering the ischemic lesions present in the sample.

Given the key role of inflammation in CE, therapies have always been based on adding steroids to statins, although there is no clear evidence of its effectiveness (2). There are also very few reports showing positive results in the use of the synthetic prostacyclin iloprost as a rescue therapy in systemic CE (2, 11, 14–16, 33, 34). Recently, prophylactic intravenous iloprost therapy has shown some effectiveness in reducing the incidence of contrast-induced nephropathy in the coronary angiography setting in patients with baseline renal insufficiency undergoing coronary intervention, a setting in which toxic ischemic damage is the leading pathogenic event to renal cells (17).

In the 80s and 90s, scientific literature put great emphasis on the prostacyclin system and on the use of prostacyclin analogs in kidney disease (14, 35, 36). The main application field was ischemic injury, but there are also some reported experiences in the field of transplantation. In fact, pretransplant graft perfusion or administration of iloprost in the early days post-transplant led to some benefits in cases of DGF and of cyclosporin-induced toxicity (18, 35, 37–40).

Regarding CE in kidney transplantation, there are no reports exploring the effectiveness of PGI_2_ agonism.

Iloprost is an analog to PGI_2_ that exerts different effects both on the vascular wall and blood cells. Acting directly on endothelial cells, smooth muscle, and adventitia, it stimulates angiogenesis, endothelial cell integrity, and relaxation of smooth muscle cells. Moreover, PGI_2_ has inhibitory effects on the activation of endothelial cells and on the proliferation and migration of smooth muscle cells (41–43). PGI_2_ acts on leukocytes stimulating the production of anti-inflammatory cytokines and inhibiting the release of IL-1, tumor necrosis factor (TNF)-α, and interferon (IFN)-γ. PGI_2_ also regulates macrophage functions, promoting their anti-inflammatory profile (44, 45). Effects on the inhibition of platelet aggregation have also been described (20, 43).

The acute continuous iloprost therapy we administered to our patients may have partially counteracted the necroinflammation and vasoconstriction caused by the emboli through vasodilatation, the inhibition of IL-1 and TNF, and the production of other cytokines; in combination with high steroid doses commonly used in the early post-transplant phases, iloprost may have strengthened the positive effects that the reduction of oxidative damage exerts on the outcome of the transplant (46).

Our cases are a good example of increasingly common complications related to detrimental vascular characteristics of grafts and recipients. Moreover, in the case of transplantation, this phenomenon could be restricted to the graft, without the occurrence of peripheral or systemic lesions. Since the embolization could be patchy, the pathognomonic lesion could be invisible in the histologic sample, making the final diagnosis even more difficult. It is important to note that the only lesion seen at biopsy could be ATN associated with an inflammatory infiltrate, easily attributable to cellular rejection (2).

## Conclusions

Acute post-transplant CE seems to be increasingly diagnosed in patients with severe atherosclerosis and ECD donors. In the context of transplantation, diagnosis can be difficult since CE can be limited to the graft and the histology can be confused with cellular rejection. As prompt treatment can help in reducing the risk of PNF and in the recovery of function, CE should always be suspected in cases of persistent DGF or acute cellular rejection not responding to therapy. Iloprost, with its vasodilator and anti-inflammatory effects, could potentially act on the molecular pathways activated by cholesterol crystals; it is our opinion that prompt intravenous therapy with iloprost, added to statins and steroids, has accelerated the good outcome of the two patients whose cases we have described in this report.

Of course, the effectiveness of iloprost infusion in hindering the inflammatory and ischemic cascades induced by CE in the immediate post-transplant setting should be investigated in depth, especially considering that prompt intervention is essential. Larger case control studies and clinical trials are needed to prove the causality between iloprost administration and the improvement of kidney function and investigate when prompt intervention is essential.

## Data Availability Statement

The datasets generated for this study are available on request to the corresponding author.

## Ethics Statement

Ethical review and approval was not required for the study on human participants in accordance with the local legislation and institutional requirements. The patients/participants provided their written informed consent to participate in this study.

## Author Contributions

VCo and GC contributed to conception, design of the work, analysis, and interpretation of data. MR, VA, FO, AA, IC, and VCu contributed to the acquisition of data for the work. GL revising it critically for important intellectual content. All the authors provide approval for publication of the content.

### Conflict of Interest

The authors declare that the research was conducted in the absence of any commercial or financial relationships that could be construed as a potential conflict of interest.
